# Aβ degradation—the inside story

**DOI:** 10.3389/fnagi.2014.00229

**Published:** 2014-08-26

**Authors:** Malcolm A. Leissring

**Affiliations:** Institute for Memory Impairments and Neurological Disorders (UCI MIND), University of California, IrvineIrvine, CA, USA

**Keywords:** β-amyloid, Alzheimer disease, proteases, degradation, intracellular space

Two decades have passed since the discovery of the first proteases that degrade the amyloid β-protein (Aβ) (Roher et al., 1994; Turner et al., 2004), the primary constituent of the amyloid plaques that characterize Alzheimer disease (AD) (Selkoe, 2000). While significant progress has been made, this is an appropriate juncture to reflect on what has been accomplished and ask which research directions are most likely to bear fruit going forward. Herein, I argue that a renewed focus on intracellular Aβ-degrading proteases (AβDPs) is a highly promising direction for future studies, one that is not only likely to advance our understanding of the fundamental molecular pathogenesis of AD, but also to critically inform the development of effective therapies for use clinically.

To date, most studies of AβDPs have focused predominantly on proteases that act extracellularly (LaFerla et al., 2007; Saido and Leissring, 2012; Leissring and Turner, 2013). This is not surprising—Aβ is, after all, a secreted peptide, and amyloid plaques form extracellularly. However, there is a growing body of evidence implicating intracellular pools of Aβ in the pathogenesis of AD (Saido and Leissring, 2012; Leissring and Turner, 2013). Generally speaking, it has been challenging to study specific pools of Aβ in conventional animal models of AD, since most models rely upon overexpression of the β-amyloid precursor protein (APP), which necessarily increases the levels of all pools of Aβ simultaneously. The study of AβDPs, by contrast, offers a unique window into the pathogenic role of Aβ, in no small part because individual AβDPs have unique subcellular localizations and pH profiles, which can be exploited to selectively target different pools of Aβ (e.g., extracellular, lysosomal, etc.) (Leissring and Turner, 2013). This can be readily achieved by overexpression, genetic deletion or pharmacological manipulation of appropriate AβDPs, either alone or in tandem with APP overexpression.

There is a surprisingly long list of reasons to focus particular attention on intracellular AβDPs. First and foremost is the fact that the production of Aβ occurs intracellularly. Aβ is produced from APP by the successive action of two proteases, known as β-secretase—or β-site APP cleaving protease 1 (BACE1)—and γ-secretase, an intramembraneous complex of four proteins, with presenilin-1 or -2 comprising the active site (De Strooper et al., 2010). Of note, β- and γ-secretase are both aspartyl proteases and, as such, require an acidic environment to effect their proteolytic activity (De Strooper et al., 2010). As a consequence, although there is some evidence for limited production of Aβ at the cell surface (Chyung et al., 2005), the vast majority of Aβ is produced intracellularly, within acidified compartments. From a therapeutic perspective, it is logical to study AβDPs that are located closest to the sites of Aβ production, as they are best positioned to efficiently regulate Aβ levels, including Aβ in the extracellular space.

Second, somewhat counter-intuitively, the fraction of Aβ amenable to degradation (i.e., non-aggregated) is primarily located intracellularly, not extracellularly as is widely assumed. In the human brain, the extracellular space comprises ~5% of the total volume (Wyckoff and Young, 1956). By contrast, intracellular compartments contiguous with the extracellular space (e.g., ER, Golgi, endosomes, lysosomes, etc.) make up an estimated 17% of total cell volume (Alberts et al., 2008). Significantly, this figure ignores the many other intracellular compartments where Aβ has been detected (e.g., mitochondria, cytosol). The reason this point is not more widely appreciated may stem from the fact that extracellular/intracellular volume ratio is dramatically reversed in cultured cells—where, not coincidentally, most known AβDPs were discovered and many studies of Aβ metabolism were performed.

Third, the fraction of Aβ present in the extracellular space is to a large degree bound to carrier proteins, notably apolipoproteins E and J (ApoE, ApoJ) (Bu, 2009). When bound to ApoE or other proteins, Aβ is protected from clearance by AβDPs. Moreover, a principal function of these same molecules is, in fact, to transport Aβ to intracellular sites for degradation (Bu, 2009; Fuentealba et al., 2010).

Fourth, intracellular Aβ is far more prone to aggregation than extracellular Aβ, because Aβ aggregation is dramatically accelerated under acidic conditions (Su and Chang, 2001). Not only is newly synthesized Aβ produced within acidic compartments, but as mentioned, extracellular Aβ is also transported to lysosomes by ApoE and other Aβ-binding proteins (Bu, 2009). This point is key, because aggregated Aβ is far less amenable to clearance by proteolytic degradation or other means.

In addition to the preceding, largely theoretical, arguments for focusing on intracellular Aβ degradation, there is a compelling body of empirical evidence that is supportive, as well. In animal studies, overexpression or genetic deletion of extracellular AβDPs have had the expected effects on cerebral Aβ levels and amyloid plaques (Saido and Leissring, 2012). For instance, recombinant neprilysin (Park et al., 2013), administered either directly to brain or via peripheral administration by way of a brain-targeting domain (Spencer et al., 2014), not only lowered brain Aβ levels, but also ameliorated the learning and memory deficits present in APP transgenic mice. Similarly, neuronal overexpression of neprilysin in APP transgenic mice, achieved via peripheral administration of an adeno-associated viral vector designed to target neurons, achieved similar results (Iwata et al., 2013). However, the full complement of favorable biochemical and behavioral outcomes of the latter studies have not been uniformly observed. For example, one highly notable study (Meilandt et al., 2009) investigated a transgenic mouse model with high-level overexpression of neprilysin (Leissring et al., 2003). While neprilysin overexpression was found to markedly reduce extracellular Aβ levels and, in fact, to completely eliminate all amyloid plaque formation in a robust APP transgenic mouse model (Mucke et al., 2000), levels of oligomeric forms of Aβ—an especially neurotoxic species strongly implicated in the AD pathogenesis—were found to be unchanged in these animals (Meilandt et al., 2009). Crucially, neprilysin overexpression failed to mitigate the learning and memory defects present in the AD mouse model used in this study (Meilandt et al., 2009). The marked difference in the outcomes of these aforementioned studies does not have a ready explanation, but may be attributable to the different forms of neprilysin examined (i.e., soluble vs. membrane-bound), to the extent of overexpression achieved, or to the particular animal model employed. Nevertheless, the latter study does demonstrate that it is possible to completely eliminate all extracellular amyloid deposition, while having no effect on learning and memory and while leaving intact a critical pool of intracellular Aβ.

Conversely, emerging evidence suggests that selective manipulation of intracellular AβDPs can yield the hoped-for effects on behavioral outcomes. For instance, pharmacological enhancement of lysosomal Aβ degradation was found to effectively reverse the defects in cognitive performance as well as synaptic composition present in two AD mouse models (Butler et al., 2011). Notably, the enhancement of lysosomal degradation also resulted in a lowering of extracellular Aβ deposition (Butler et al., 2011).

Results such as these imply that the extracellular pool of Aβ may be much less consequential than has been widely assumed. As radical as it may sound, these findings suggest that extracellular Aβ deposition may in fact constitute a somewhat of an “epiphenomenon” obscuring more material events going on at intracellular sites (Leissring and Turner, 2013). This conclusion is bolstered by other experimental paradigms, particularly studies of the effects of overexpressing Aβ alone within different subcellular compartments. For example, expression of Aβ exclusively in the extracellular space led to amyloid plaque formation (McGowan et al., 2005; Kim et al., 2013), as expected, but resulted in no significant effects on learning and memory (Kim et al., 2013). In marked contrast, overexpression of Aβ in the cytosol has been shown to be profoundly toxic in cultured cells (Zhang et al., 2002) and, *in vivo*, resulted in profound neurodegeneration (LaFerla et al., 1995)—a feature that is notably absent from many other animal models of AD.

The possible pathogenic role of intracellular Aβ—and, indeed, its very existence—has been a subject of controversy for several decades (LaFerla et al., 2007). A large number of studies have identified Aβ within non-canonical compartments, including the cytosol and mitochondria (LaFerla et al., 2007). But evaluating the veracity of these claims has been complicated by the difficulty in reliably detecting the relatively small amounts of putative intracellular Aβ—particularly against a background of abundant Aβ present extracellularly and within the endolysosomal pathway and other canonical intracellular compartments. Nevertheless, in my opinion, the evidence supporting the idea that intracellular Aβ may be particularly pathogenic is sufficiently compelling to merit further investigation.

The study of intracellular AβDPs may be an effective way to finally resolve the controversy surrounding both the existence of and the pathogenic role of intracellular Aβ. By virtue of their distinctive subcellular localizations and pH optima, AβDPs constitute powerful tools for manipulating different pools of Aβ and, thereby, gaining fresh insight into their potential involvement in the pathogenesis of AD (Leissring and Turner, 2013). AβDPs are present in a diverse range of subcellular compartments: insulin-degrading enzyme (IDE) in cytosol (Roth, 2004), IDE and presequence peptidase in mitochondria (Leissring et al., 2004; Falkevall et al., 2006), BACE-2 and endothelin-converting enzymes 1- and -2 in endosomes (Eckman et al., 2001; Abdul-Hay et al., 2012), and in cathepsins B and D in lysosomes (Gan et al., 2004; Leissring et al., 2009) (see Saido and Leissring, 2012; Leissring and Turner, 2013 for comprehensive reviews). Collectively, these AβDPs represent a diverse set of experimental tools for selectively manipulating different pools of Aβ. Because AβDPs degrade other substrates and also subserve different physiological functions, studies based on them cannot be expected to constitute perfectly “clean” tests of the importance of different pools of Aβ. Nevertheless, comparison of the consequences of genetic deletion and/or overexpression of spatially distinctive AβDPs is virtually certain to offer additional insight into the roles that different pools of intracellular Aβ do or do not play in AD pathogenesis. To the extent possible, comparative studies of this type would ideally be conducted within the same animal model and utilize the same behavioral and biochemical outcomes. Additionally, the use of an animal model that harbors human tau might help to elucidate the mechanistic link between Aβ accumulation and tauopathy, one of the most important unresolved questions in AD research.

In conclusion, there is a compelling theoretical and empirical rationale for the field to undertake a renewed focus on intracellular AβDPs. The knowledge we can expect to derive from the study of extracellular AβDPs appears to be, at best, approaching an asymptote and, at worst, revealing that extracellular pools of Aβ may not be involved in the pathogenesis of AD to the extent so widely assumed for so long. The study of intracellular AβDPs, by contrast, seems poised to yield insights into questions that are not merely academic or theoretic, but highly practical—for example, the relative merits of immunotherapies, which only target extracellular Aβ, versus secretase inhibitors or modulators, which affect intracellular Aβ as well. Considering the growing interpersonal, financial and societal impact of AD, and the current lack of therapies, it is wise to pursue any and all avenues that may lead to effective treatments, and the study of intracellular AβDPs seems an especially promising direction for future investigation.

## Conflict of interest statement

The author declares that the research was conducted in the absence of any commercial or financial relationships that could be construed as a potential conflict of interest.
